# Acupuncture for the Treatment of Chronic Rhinosinusitis: A PRISMA-Compliant Systematic Review and Meta-Analysis

**DOI:** 10.1155/2022/6429836

**Published:** 2022-08-31

**Authors:** Boram Lee, Chan-Young Kwon, Man Young Park

**Affiliations:** ^1^KM Science Research Division, Korea Institute of Oriental Medicine, Daejeon 34054, Republic of Korea; ^2^Department of Oriental Neuropsychiatry, Dong-Eui University College of Korean Medicine, Busan 47227, Republic of Korea; ^3^Digital Health Research Division, Korea Institute of Oriental Medicine, Daejeon 34054, Republic of Korea

## Abstract

**Background:**

Chronic rhinosinusitis (CRS) is a highly prevalent disease associated with poor quality of life. In this paper, we appraised the role of acupuncture in the treatment of CRS.

**Methods:**

Electronic databases were searched for randomized controlled clinical trials (RCTs) that examined the role of acupuncture in CRS. The primary outcome measures included posttreatment CRS severity, as measured by the Visual Analogue Scale (VAS) and Total Effective Rate (TER). The risk of bias and quality of evidence were evaluated according to the Cochrane Collaboration's risk-of-bias tool and GRADE tool, respectively.

**Results:**

Evidence from the RCTs (*n* = 10) suggested that acupuncture as a monotherapy or adjunctive therapy to conventional treatment was associated with significant improvements in VAS, TER, and quality of life when compared with conventional treatments for CRS. However, there was a similar incidence of adverse events. The risk of bias was unclear and the quality of evidence for each finding was generally moderate to low.

**Conclusions:**

Acupuncture as a stand-alone or adjunctive treatment for CRS was associated with clinical symptom improvement and better quality of life, without any risk for serious adverse events. However, the high clinical heterogeneity of the included RCTs and overall moderate-to-low quality of evidence necessitates rigorous, well-designed trials to confirm these findings. *Trial Registrations*. This trial is registered with PROSPERO (no. CRD42021292135).

## 1. Introduction

Chronic rhinosinusitis (CRS) is diagnosed when at least two of the symptoms (nasal drainage, nasal obstruction, facial pain/pressure, and hyposmia/anosmia) are present for at least 12 consecutive weeks. Additionally, diagnosis is based on a comprehensive physical examination or objective radiographic findings [1]. The estimated prevalence of CRS is 3.0–6.4% in the general population [1]. It places a tremendous burden on healthcare services and is associated with severe morbidity and poor health-related quality of life [2]. A survey conducted by a tertiary care sinus center in Canada showed that the total out-of-pocket expenses incurred due to CRS are $614.06 per patient per year [3].

The goal of CRS treatment is to provide symptomatic relief and improve the health-related quality of life. Conventional treatments for CRS include intranasal saline and medications, including corticosteroids, antibiotics, and leukotriene receptor antagonists. Surgical treatment may be considered if there is no improvement with medication [1]. However, evidence for the efficacy of medications, especially antibiotics, is limited [4]. Moreover, the possible adverse effects, drug resistance, and recurrence rates are high [5, 6]. The limitations of conventional treatments and chronic nature of the disease motivate patients to seek out complementary and integrative medicine (CIM) treatments, such as acupuncture, moxibustion, and herbal medicine [7, 8]. In keeping with these needs, some clinics attempt to treat refractory CRS using CIM because of the evidence that it improves the symptoms and quality of life [9].

Acupuncture, one of the representative CIMs, has long been used to treat various diseases, including CRS. According to a modified Delphi study conducted in the United States, alternative treatments for CRS, such as herbal medicines, vitamin supplementation, diet, and acupuncture, lack sufficient evidence to support their use in isolation. However, acupuncture could be considered as an adjuvant treatment due to its low risk [10]. The anti-inflammatory effects of acupuncture are mediated by a complex neuroendocrine-immunological network, which may help improve CRS [11]. Therefore, this treatment is a promising and cost-effective candidate for alleviating the disease burden associated with CRS.

Two reviews summarizing the effect of acupuncture on the treatment of CRS have been published [12, 13]; however, one was not systematic or comprehensive enough because they only searched the PubMed database and only a descriptive analysis was performed on the included studies [12]. The remaining study employed a systematic review protocol but no evaluation plan for assessing the quality of evidence on the key findings was provided [13]. The aim of this review was to provide a comprehensive overview of evidence concerning the efficacy and safety of acupuncture treatment for CRS by comprehensively searching databases and assessing the quality of evidence on major findings. Insight gathered from this review could help inform policy decision-making and guide patient management by clinicians.

## 2. Methods

We performed the research in accordance with the Preferred Reporting Items for Systematic Reviews and Meta-Analyses (PRISMA) guidelines [14]. Ethical approval was not required for this systematic review because we used publicly accessible documents as evidence.

### 2.1. Inclusion and Exclusion Criteria

#### 2.1.1. Type of Study Design

Parallel-group, randomized controlled clinical trials (RCTs) evaluating the effect and safety of acupuncture for CRS were included. To lower the risk of potential bias, crossover trials were excluded. There were no restrictions applied to the publication language (e.g., English, Korean, Chinese, or Japanese were included).

#### 2.1.2. Type of Participants

We included trials involving patients with CRS without limitation on age, sex, or race. However, studies in which a diagnosis of CRS could not be confirmed were excluded.

#### 2.1.3. Type of Treatment and Control Interventions

Studies using any type of acupuncture-related therapies, such as manual acupuncture, electroacupuncture, and moxibustion, were included. We excluded studies that did not provide information regarding the acupuncture points used. Sham acupuncture, no medical treatment, and conventional Western medicine treatments, such as antibiotics and antihistamines, were allowed as control interventions. However, studies using other CIM interventions as a control intervention were excluded. Studies involving acupuncture combined with conventional treatments were also included if the use of conventional treatments was equal across the treatment and control groups.

#### 2.1.4. Type of Outcome Measures

The primary outcome measure of interest was posttreatment CRS severity. This was measured using the 0–10 cm Visual Analogue Scale (VAS) and Total Effective Rate (TER). The VAS is a subjective outcome to measure the severity of disease-related symptom; higher scores indicate greater severity [15]. The TER is defined according to specific evaluation criteria, including symptomatic improvement and the improvement rates of other quantified outcomes.

Secondary outcome measures of interest included (1) posttreatment health-related quality of life, measured using instruments like the Sino-Nasal Outcome Test (SNOT)-20 [16]; (2) posttreatment endoscopic scores like the Lund-Kennedy endoscopic scores [17]; (3) posttreatment computed tomography (CT) scan scores like the Lund-Mackay CT scores [18]; (4) CRS recurrence rates; and (5) the incidence of adverse events (AEs) during the study period. The SNOT-20 is one of the most widely used quality-of-life tools for sinus diseases and evaluates 20 items using a 0–5 scale; higher scores indicate poorer quality of life [16]. The Lund-Kennedy score assesses the bilateral presence and severity of edema, scarring and crusting of the nasal mucosa, discharge, and polyps (from 0 to 2), for a total score range of 0–20; higher scores indicate higher severity [17]. The Lund-Mackay CT score assesses 6 bilateral areas (maxillary, anterior ethmoids, posterior ethmoids, sphenoid, frontal, and ostiomeatal complex) of sinus opacification (from 0 to 2), for a total score range of 0–24; higher scores indicate greater sinus opacity [18].

### 2.2. Information Sources and Search Strategy

The following 14 databases were searched for studies published from their inception dates: Medline, EMBASE, Cochrane Central Register of Controlled Trials, Allied and Complementary Medicine Database, Cumulative Index to Nursing and Allied Health Literature, Oriental Medicine Advanced Searching Integrated System, Korean Studies Information Service System, Korean Medical Database, Research Information Sharing Service, ScienceON, China National Knowledge Infrastructure, Wanfang Data, Chongqing VIP, and CiNii. The initial search date was July 6, 2021; we conducted another search on April 7, 2022, to collect more up-to-date evidence. The reference lists of the included studies and trial registries, including the international clinical trials registry platform of the World Health Organization, were reviewed to include as many eligible studies as possible. In addition to journal publications, we considered grey literature, including degree theses. This search strategy was developed through consultation with systematic review experts. The search strategies and corresponding search results for each database are summarized in Supplementary File 1.

### 2.3. Study Selection and Data Extraction

The studies selected following our search of the databases and other sources were imported into EndNote 20 and duplicates were removed. The titles and abstracts of the remaining studies were then reviewed for the first inclusion. The full-text versions of all eligible studies were retrieved and reviewed for final inclusion. Study selection was conducted by one researcher (BL), while another researcher (CYK) reviewed the results. Disagreement was resolved by discussion with a third researcher (MYK). The following information was extracted from the included studies and documented in a standardized Excel 2016 form: basic study information, sample size, participant characteristics, treatment and control intervention, outcome measures, results, and information used to assess the risk of bias were recorded. We referred to the Standards for Reporting Interventions in Clinical Trials of Acupuncture (STRICTA) checklist [19] to extract detailed information regarding acupuncture treatment for each included study. Two researchers (BL and CYK) independently conducted the data extraction; any disagreement was resolved by discussion with a third researcher (MYP). We contacted the authors of the included studies via e-mail wherever possible, if the data were ambiguous.

### 2.4. Risk of Bias Assessment

We assessed the risk of bias of the included studies using the Cochrane Collaboration's risk-of-bias tool [20]. This instrument includes the domains of random sequence generation, allocation concealment, blinding of participants, personnel, and outcome assessors, completeness of outcome data, selective reporting, and other biases. Each domain was assessed as “low risk,” “unclear risk,” or “high risk” of bias by two researchers (BL and CYK); consensus was reached through discussions between them if there was any disagreement.

### 2.5. Data Analysis and Synthesis

Descriptive analysis of the details of all included studies was performed. Meta-analysis was conducted for our primary and secondary outcomes using the Review Manager (RevMan) software package (version 5.4). Continuous and binary outcomes were presented as the mean difference (MD) and risk ratio (RR) along with 95% confidence intervals (CIs). Heterogeneity among the studies was evaluated using the *χ*^2^ test and the *I*^2^ statistics. We considered an *I*^2^ value ≥ 50% and ≥75% as indicative of substantial and considerable heterogeneity, respectively. Owing to the lack of precision of the estimates of between-study variance, a fixed-effects model was used if the heterogeneity was not significant (*I*^2^ < 50%), or if the number of studies included in the meta-analysis was small [21]. Otherwise, a random-effects model was used. To determine the cause of heterogeneity, a subgroup analysis was conducted according to the type of acupuncture treatment (manual acupuncture, electroacupuncture, or moxibustion). A sensitivity analysis was conducted to identify the robustness of the meta-analysis by excluding studies without any details of the random sequence generation method.

### 2.6. Quality of Evidence Assessment

The quality of evidence for key findings was assessed using the Grading of Recommendations, Assessment, Development, and Evaluations (GRADE) tool as “very low,” “low,” “moderate,” or “high” [22]. The risk of bias, inconsistency, indirectness, imprecision, and publication bias were assessed for each finding using GRADEpro (https://gradepro.org/). The assessment was performed by one researcher (BL), while another researcher (CYK) reviewed the results. Any disagreements were resolved via discussion between them.

## 3. Results

### 3.1. Study Selection

**Figure 1:**
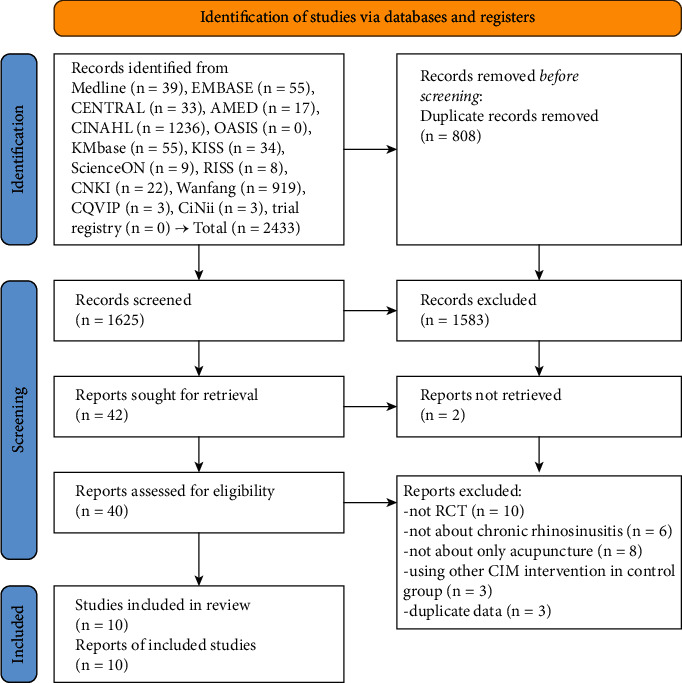
A PRISMA flow diagram of the literature screening and selection processes.

The initial search of the electronic databases identified 2433 studies; no records were identified while searching the trial registry and citations. After removing 808 duplicates, the titles and abstracts of 1625 studies were screened, and 1583 studies were excluded. The full texts of the remaining 42 studies were retrieved; two articles were not retrieved. Therefore, the final full-text publications of 40 available studies were assessed for eligibility. After excluding studies other than RCTs (*n* = 10), those not about CRS (*n* = 6), those including treatments other than acupuncture (*n* = 8), those using other CIM interventions in the control group (*n* = 3), and those using duplicate data (*n* = 3), a total of 10 studies [23–32] were included in this review (Figure 1 and Supplementary File 2).

### 3.2. Study Characteristics

The studies included in this review (*n* = 10) were published between 2005 and 2022. Nine studies were conducted in China and one study was conducted in Norway [30]. Five studies [24, 25, 27, 29, 31] compared acupuncture to conventional treatment and four studies [23, 26, 28, 32] compared acupuncture plus conventional treatment to conventional treatment alone. One study [30] was a three-arm clinical trial comparing acupuncture, sham acupuncture, and conventional treatment. In most studies, CRS was diagnosed based on clinical symptoms, nasal endoscopy, and/or sinus CT. Four studies specified the pattern identification of participants. There was one study of Qi-blood deficiency complicated by stasis [24], lung Qi-deficiency cold [25], and wind and cold damage [27]. One study [30] recruited participants corresponding to four pattern identifications (retention of damp in the Yangming channel, damp combined with heat, retention of phlegm in the Shaoyang channel, and liver fire), and acupuncture treatment was performed accordingly. Four studies [23, 28, 30, 32] reported that they had obtained permission from an institutional review board before conducting the study, and five studies [23–25, 28, 32] reported that informed consent was obtained from the participants. Four studies [25, 29, 30, 32] mentioned funding sources, all of which were provided by the state or local province.

Five studies [24, 28–31] used manual acupuncture, two studies [26, 32] used electroacupuncture, two studies [25, 27] used moxibustion, and one study [23] used transcutaneous electrical acupoint stimulation (TEAS). For the acupuncture points, the most frequently used was LI4 [23, 24, 28, 30–32], followed by EX-HN3 [24, 25, 27, 28, 31] and LI20 [23–25, 28, 31] in five studies and GV20 in three studies [24, 28, 31]. In most studies, the needle retention time was 20–30 min and treatment frequency was once or twice daily. The treatment duration varied between 1 and 70 days, with a mean duration of 2 weeks (Tables 1 and 2).

### 3.3. Risk of Bias Assessment

**Figure 2:**
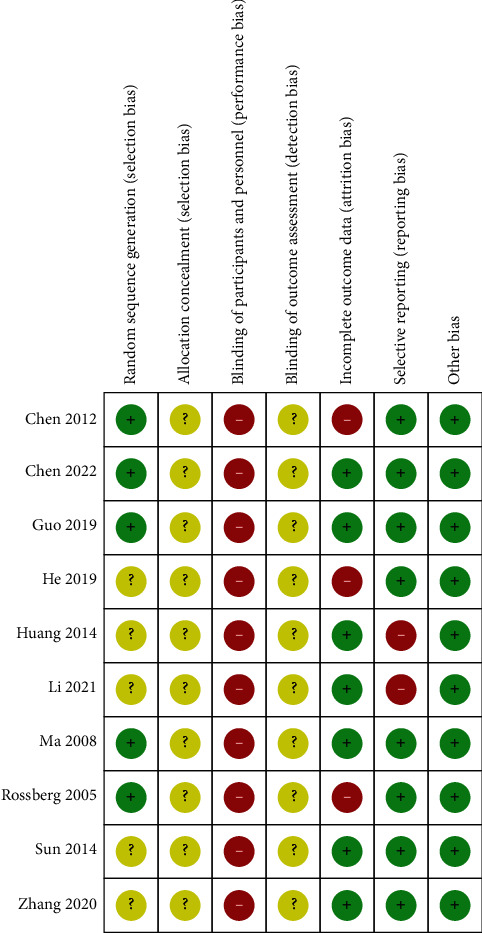
Risk-of-bias summary for all included studies. Low, unclear, and high risk, respectively, are represented by the following symbols: “+,” “?,” and “−.”

As five studies [23–25, 29, 30] used appropriate random sequence generation methods, the risk of selection bias was low. No studies reported information regarding allocation concealment, blinding of participants, personnel, or outcome assessors. Three studies [24, 26, 30] that performed only per-protocol analysis, and two studies [27, 28], which only reported TER without corresponding raw data, were evaluated as having a high risk of attrition bias and reporting bias, respectively. All studies included in this review mentioned statistical baseline homogeneity between the treatment and control groups. The risk of other biases was low (Figure 2).

### 3.4. Acupuncture versus Conventional Treatment

**Figure 3:**
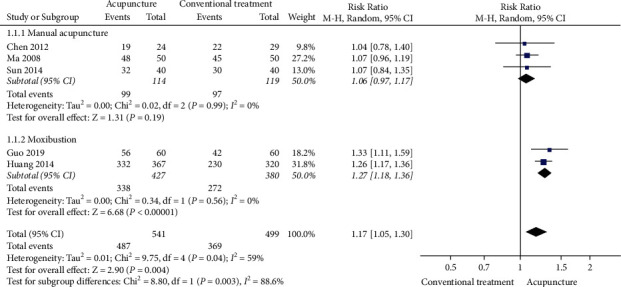
Forest plot of total effective rate: acupuncture versus conventional treatment.

**Figure 4:**
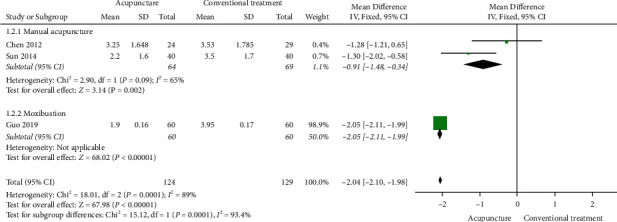
Forest plot of visual analogue scale: acupuncture versus conventional treatment.

A meta-analysis showed that acupuncture resulted in a higher TER than conventional treatment (5 studies, RR 1.17, 95% CI 1.05 to 1.30, *I*^2^ = 59%; Figure 3). Subgroup analysis stratified according to the type of acupuncture significantly lowered the statistical heterogeneity, although the significance of acupuncture was maintained only in the case of moxibustion. Moreover, sensitivity analysis, performed by excluding studies without any details of the random sequence generation method, showed that the significance of acupuncture was maintained only in the case of moxibustion. In addition, the severity of CRS measured by VAS (3 studies, MD −2.04, 95% CI −2.1 to −1.98, *I*^2^ = 89%; Figure 4), the quality of life measured by SNOT-20 (1 study, MD −3.48, 95% CI −3.58 to −3.38), Lund-Kennedy endoscopic scores (1 study, MD −1.25, 95% CI −1.82 to −0.68), and Lund-Mackay CT scores (1 study, MD −1.23, 95% CI −1.8 to −0.66) were all significantly improved in the acupuncture group when compared with the conventional treatment group. The statistical significance was maintained when sensitivity analysis was performed by excluding studies without any details of the random sequence generation method. There was no difference between the two groups in CRS recurrence rates after 6 months (1 study, RR 0.55, 95% CI 0.23–1.32). One study [24] reported AEs: one case of mild fever in the manual acupuncture group and two cases of nausea and vomiting in the ibuprofen group. The incidence of AEs was similar between the two groups (1 study, RR 0.60, 95% CI 0.06–6.26; Table 3).

Two studies [24, 31] examined patients with CRS after functional endoscopic sinus surgery (FESS) and compared manual acupuncture with sustained-release ibuprofen treatment. Both studies found improvements in total and present pain intensity assessed using the short-form McGill pain questionnaire in the acupuncture group when compared with the ibuprofen group. Rossberg et al. [30] compared manual acupuncture to conventional treatment, including the use of a local vasoconstrictor agent and a 0.9% sodium chloride solution, oral corticosteroids, and antibiotics for patients with CRS without nasal polyps or pansinusitis. There were no significant differences in these outcomes after treatment between the two groups.

### 3.5. Acupuncture Plus Conventional Treatment versus Conventional Treatment Alone

The CRS severity measured by TER was significantly higher after treatment in the acupuncture plus conventional treatment group compared to the conventional treatment group (2 studies, RR 1.25, 95% CI 1.11–1.40, *I*^2^ = 0%), regardless of the type of acupuncture used. In addition, the VAS for pain was significantly reduced in the acupuncture plus conventional treatment group (2 studies, MD −1.07, 95% CI −1.27 to −0.87, *I*^2^ = 98%). Statistical significance was maintained when sensitivity analysis was performed by excluding studies without any details of the random sequence generation method. Zhang et al. [32] targeted CRS populations in whom conservative treatment was not effective and who had no contraindications to FESS. They compared electroacupuncture plus FESS under intravenous general anesthesia combined with endotracheal intubation to FESS alone. The results showed that the need for additional doses of propofol and fentanyl during surgery was significantly lower in the acupuncture group. However, there were no significant differences between the two groups in terms of heart rate, mean arterial pressure, bleeding during operation, and operation time. He et al. [26] compared electroacupuncture plus conventional treatment (amoxicillin and topic furosemide nasal drops) with conventional treatment alone. The authors found a significant improvement in CRS symptoms in the acupuncture group; however, there were no significant differences between the groups in recurrence rates after 1- and 3-month follow-up. The authors [26] reported that there was only 1 case of hematoma in the electroacupuncture group; there was no significant difference in AEs between the two groups (1 study, RR 3.09, 95% CI 0.13–73.19; Table 3).

### 3.6. Acupuncture versus Sham Acupuncture

Rossberg et al. [30] compared manual acupuncture and sham acupuncture (minimal acupuncture at non-acupoints) for patients with CRS without nasal polyps or pansinusitis. After treatment, there were no differences compared with baseline in both groups in sinus soft tissue swelling assessed by CT, quality of life, and EuroQol VAS; there were no statistical comparisons reported between the groups. The authors reported small bleeds at the needle insertion site, nerve pain, and dizziness as AEs; their incidence was similar between groups (1 study, RR 1.50, 95% CI 0.63–3.53; Table 3).

### 3.7. Quality of Evidence

The overall quality of evidence was generally “moderate” or “low,” and high-quality evidence was lacking. The main reason for downgrading the quality of evidence was the risk of bias of the included RCTs and the imprecision of the meta-analyzed results due to small sample sizes and wide CIs (Table 3).

## 4. Discussion

### 4.1. Summary of Evidence

In this review of RCTs (*n* = 10), we evaluated the effect and safety of acupuncture for CRS. A meta-analysis showed that acupuncture as monotherapy or adjunctive therapy to conventional treatment was associated with significantly higher TER and lower VAS than conventional treatment alone. Interestingly, subgroup and sensitivity analyses showed that moxibustion as a monotherapy was associated with significantly greater TER than conventional treatment. In several studies, compared with conventional treatment, moxibustion as a monotherapy was associated with more favorable posttreatment outcomes, as measured by SNOT-20, Lund-Kennedy endoscopic, and Lund-Mackay CT scores. In addition, manual acupuncture and TEAS add-ons to conventional treatment were associated with significantly higher TER and lower VAS than conventional treatment alone. There was no difference in the incidence of AEs between the acupuncture and control groups. The methodological quality of the included studies was suboptimal. In particular, the risk of bias for selection, performance, and detection was evaluated as unclear or high. The quality of evidence of the main findings was mostly moderate or low, as evaluated by GRADE.

### 4.2. Implications of the Results

CRS is a chronic illness associated with a significant disease burden and poor quality of life [3]. Limitations of conventional treatments have led to a significant interest in CIM as an alternative treatment modality [7, 8]. In this evidence-based systematic review, we examined the role of acupuncture in the treatment of CRS. It was anticipated that this could help inform clinical decision-making. The findings of the review suggested that acupuncture is associated with better TER and VAS for the treatment of CRS. However, there were significant methodological limitations across these studies, and the quality of evidence was moderate-to-low. Nevertheless, there is little high-level evidence to support current treatment protocols for CRS [33]; therefore, acupuncture should be considered a promising treatment candidate.

The pathogenesis of CRS is not fully understood; however, it may involve chronic inflammation, alterations in mucociliary clearance, abnormalities in the sinonasal epithelial cell barrier, and tissue remodeling [34–36]. According to a preclinical animal study, manual acupuncture and moxibustion can reduce the inflammatory response by decreasing the expression of thymic stromal lymphopoietin (TSLP) protein in the nasal sinus mucosa [37]. TSLP, the master regulator of Th2-mediated inflammation, correlates with the inflammatory markers of Th2 inflammation in sinus tissue [38]. Moreover, adjuvant electrostimulation therapy can improve CRS symptoms by affecting inflammation [39], supporting the applicability of electroacupuncture found in this review.

In addition, the findings of this review highlight the frequent use of certain acupuncture points, including LI4, for the treatment of CRS. Interestingly, LI4 is a frequently used acupuncture point for the treatment of allergic rhinitis [40]. This point is linked to the activity of a specific brain region responsible for the orofacial area [41]; its stimulation is associated with an anti-inflammatory response [42]. In addition, although not used in the studies included in this review, another recent systematic review reported that acupuncture at the sphenopalatine ganglion acupoint may be an effective alternative therapy for patients with allergic rhinitis [43]. Hence, the impact of this strategy on CRS should be considered in further studies. As pointed out in a recent review, the current level of evidence is considered insufficient to routinely recommend acupuncture for CRS. However, it can be considered a useful and promising candidate for CRS treatment, especially as an adjunct to conventional therapy [12]. Nonetheless, rigorous research is needed to elucidate the clinical effects and underlying mechanisms of acupuncture treatment for CRS.

### 4.3. Potential Mechanisms of Action of Acupuncture for CRS

Although the underlying treatment mechanism of acupuncture for CRS requires further elucidation, one possible explanation is the anti-inflammatory effects of acupuncture via antihistamine and the downregulation of proinflammatory cytokines, chemokines, and neuropeptides [44]. A recent study has reported that electroacupuncture suppresses the decrease in the expression of interferon-*γ*, alleviates mucosal damage caused by inflammation, and shows a synergistic beneficial effect when combined with interleukin-10 (i.e., overexpression) in a mouse model with chronic sinusitis [45]. Moreover, acupuncture combined with moxibustion in CRS mice may reduce the inflammatory response by downregulating the expression of TSLP protein in nasal sinus mucosa [37]. This anti-inflammatory effect is thought to be obtained by proximal acupuncture (e.g., intranasal acupuncture) [46, 47] or distal acupuncture [48]; however, the mechanism of each is suspected to be different. In addition to its anti-inflammatory and immunomodulatory effects, stimulation of specific acupoints may help to improve symptoms in patients with CRS by modulating sympathetic excitability. For example, acupuncture targeting the sphenopalatine ganglion improves nasal ventilation in healthy volunteers. This is mediated by increased sympathetic nerve excitability, which decreases nasal nitric oxide levels, attenuates nitric oxide-induced vasodilation, and decreases nasal congestion [49, 50]. However, the effect of acupuncture on CRS requires further elucidation.

### 4.4. Limitations and Suggestions for Future Research

This study provided a comprehensive and critical review of acupuncture treatment for CRS and assessed the quality of evidence for key findings; however, the following limitations should be considered. First, because of the limited number of studies reviewed, most meta-analysis results were based on one to three studies. Moreover, since the methodological quality of the included studies was not high, future robust high-quality studies should be conducted. The results of those future studies may invalidate or validate our findings. Many patients with CRS are interested in CIM modalities [7, 8], including acupuncture; therefore, clinical trials on this topic should be encouraged to facilitate evidence-based decision-making in clinical settings. Second, it appears that there is no differentiated acupuncture strategy based on the classification of CRS. In general, CRS can be classified according to the presence or absence of nasal polyps and these types have different inflammatory characteristics [51]. In addition, recent findings suggest that CRS with nasal polyps can be further classified as eosinophilic and noneosinophilic [51]. The elucidation of the underlying mechanism of acupuncture for CRS could help establish an optimized treatment strategy based on the classification of CRS. Third, acupuncture, as defined in this review, included manual acupuncture, electroacupuncture, and moxibustion; therefore, we performed a subanalysis to explore the effects of each acupuncture type. However, the observed heterogeneity could not be fully explained due to an insufficient study number. The findings of this review suggest that moxibustion may be a particularly useful monotherapy option for CRS. Since moxibustion is a noninvasive treatment, it might be useful in patients who are afraid of needles or pain caused by the insertion of acupuncture needles. Finally, a funnel plot could not be generated to evaluate publication bias due to the insufficient number of included studies. Additionally, most of the included studies were conducted in China; therefore, more studies should be conducted in different countries.

## Figures and Tables

**Table 1:** General characteristics of the included studies.

Study ID	Sample size (A) : (B) (enrolled ⟶ analyzed)	Mean age (range) (yr)	CRS disease period	CRS diagnosis	Population	(A) Treatment intervention	(B) Control intervention	Outcome
Chen [24]	57 (27 : 30)⟶53 (24 : 29)	(A) 46.33 ± 15.871 (B) 36.97 ± 12.365	Not reported	Not reported	Clear pain in the nose, forehead, and head within 48 hr after FESS (VAS 4–7)	Manual acupuncture	Ibuprofen sustained-release capsule	(1) VAS (0–10) (2) TER (3) SF-MPQ total score (0–16) (4) SF-MPQ present pain intensity score (0–5) (5) Bruggman comfort scale (0–4)

Chen et al. [23]	70 (35 : 35) ⟶ 70 (35 : 35)	(A) 42.18 ± 5.30 (20–57) (B) 42.15 ± 5.78 (22–58)	Not reported	Not reported	CRS, population after FESS	Transcutaneous electrical acupoint stimulation + (B)	Routine perioperative nursing	(1) VAS (0–10) (2) Self-rating anxiety score (3) Total time of deep sleep

Guo et al. [25]	120 (60 : 60) ⟶ 120 (60 : 60)	(A) 22–61 (B) 19–63	>12 wk	Clinical symptom, nasal endoscopy, and/or sinus CT	CRS, excluded population with nasal polyps	Moxibustion	Budesonide nasal spray, roxithromycin tablets	(1) TER (2) VAS (0–10) (3) SNOT-20 (4) Lund-Kennedy endoscopic score (5) Lund-Mackay CT score

He et al. [26]	72 (36 : 36) ⟶ 65 (32 : 33)	(A) 38.23 ± 1.32 (26–71) (B) 36.87 ± 2.14 (22–67)	>12 wk (A) 13.64 ± 2.01 yr (1–26) (B) 11.64 ± 1.23 yr (1–21)	Clinical symptom, nasal endoscopy, and/or sinus CT	CRS	Electroacupuncture + (B)	Amoxicillin capsule, topical furosemide nasal drops	(1) TER (2) Symptom score (3) Recurrence after 1 mo·f/u (4) Recurrence after 3 mo f/u

Huang et al. [27]	687 (367 : 320) ⟶ 687 (367 : 320)	(A) 16–80 (B) 17–78	(A) 6 mo–25 yr (B) 3 mo–23 yr	Clinical symptom, nasal endoscopy, and sinus CT or MRI	CRS, excluded population with indications for surgery	Moxibustion + roxithromycin	Budesonide nasal spray, roxithromycin tablets	(1) TER

Li and Fu [28]	100 (50 : 50) ⟶ 100 (50 : 50)	(A) 48.79 ± 6.47 (20–70) (B) 48.25 ± 6.92 (20–70)	Not reported	Not reported	CRS	Manual acupuncture + (B)	1% ephedrine saline into the turbinate, antibiotics	(1) TER

Ma et al. [29]	100 (50 : 50) ⟶ 100 (50 : 50)	(A) 30.0 ± 11.6 (B) 29.0 ± 10.5	>3 mo (A) 14.5 ± 5.6 yr (B) 12.8 ± 4.5 yr	Clinical symptom, sinus radiology, and/or endoscopy	CRS	Manual acupuncture	Antibiotics (roxithromycin, penicillin, or cephalosporin), chlorpheniramine, furosemide nasal drops	(1) TER (2) Headache disappearance time (3) Treatment period of effective cases (4) Recurrence rate after 6 mo·f/u

Rossberg et al. [30]	55 (25 : 19 : 21) ⟶ 39 (16 : 13 : 10)	(A) 41.1 ± 14.7 (B1) 47.3 ± 13.7 (B2) 41.0 ± 13.0	>3 mo (A) 7.0 ± 7.8 yr (B1) 12.4 ± 13.5 yr (B2) 9.7 ± 10.5 yr	Clinical symptom, and sinus CT	CRS, excluded population with nasal polyps and pansinusitis	Manual acupuncture	(B1) minimal acupuncture at non-acupoints (B2) local vasoconstrictor agent (xylometazoline), 0.9% sodium chloride solution, oral corticosteroids, (if needed) cefalexin and azithromycin	(1) Sinus soft tissue swelling as assessed by CT (mm) (change) (2) Self-administered questionnaire (change) (3) SF-36 PCS (change) (4) SF-36 MCS (change) (5) Euroqol VAS (change)

Sun [31]	80 (40 : 40) ⟶ 80 (40 : 40)	(A) 37.5 ± 5.5 (19–60) (B) 37.0 ± 5.0 (18–59)	Not reported	Clinical symptom, nasal endoscopy, and/or sinus CT	CRS, population after FESS under local anesthesia	Manual acupuncture	Ibuprofen sustained-release capsule	(1) TER (2) VAS (0-10) (3) SF-MPQ total score (0–16) (4) SF-MPQ present pain intensity score (0–5)

Zhang et al. [32]	120 (60 : 60) ⟶ 120 (60 : 60)	(A) 40 (B) 38	Not reported	Clinical symptom, and sinus CT	CRS, conservative treatment is not effective and has no contraindications to FESS	Electroacupuncture + (B)	FESS under intravenous general anesthesia combined with endotracheal intubation	(1) Additional doses of propofol during operation (mg/kg) (2) Additional doses of fentanyl during operation (*μ*g/kg) (3) Heart rate during operation (per min) (4) Mean arterial pressure during operation (kPa) (5) Operation time (min) (6) Bleeding during operation (mL) (7) VAS at 6 hr after operation (0–10)

CRS: chronic rhinosinusitis; CT: computer tomography; FESS: functional endoscopic sinus surgery; MCS: mental component summary; PCS: physical component summary; SF-MPQ: the short-form McGill pain questionnaire; SF-36: the 36-item short-form survey; SNOT: sinonasal outcome test; TER: total effective rate; and VAS: visual analogue scale.

**Table 2:** Details of acupuncture methods.

Study ID	Style of acupuncture	Number of needles	Acupuncture points	Depth of insertion	Response sought	Needle stimulation	Retention time	Needle type	Number of treatment sessions	Frequency	Treatment duration	Follow-up period
Chen [24]	Manual acupuncture	13	Bilateral LI20, BL2, EX-HN5, LI4, ST36, EX-HN3, GV20, GV24	Not reported	Perform needle manipulation so that there is a sense of acupuncture	Needle manipulation every 10 min	30 min	Disposal sterile acupuncture needle (Suzhou Tianxie acupuncture equipment Co., ltd.)	1-2	Once or twice a day (if there was no effect within 4 hours after the initial acupuncture, acupuncture was performed once more.)	1 day	None

Chen et al. [23]	Transcutaneous electrical acupoint stimulation	Not applicable	Bilateral LI4, LI20	Not applicable	Slight tingling sensation	2/100 Hz, intensity 4	30 min	A low-frequency electronic pulse therapy device (Wuxi Jiajian medical instrument Co., ltd.)	4	1, 13, 25, 37 hours after surgery	37 hours	None

Guo et al. [25]	Moxibustion	Not applicable	Bilateral LI20, EX-HN3	Not applicable	Feel the local skin warm and slightly hot without burning pain, and the local skin is slightly red without blistering	Not applicable	30 min	Ginger-separated moxibustion	14	Once a day	14 days	None

He et al. [26]	Electroacupuncture	2	ST7, SI18 (CRS affected side)	ST7: 45–60 mm SI18: 30–45 mm	Deqi (soreness, numbness, heaviness or distension)	Not reported	20 min	0.3 mm × 75 mm filiform acupuncture needle	10	5 times a week	2 weeks	1, 3 months

Huang et al. [27]	Moxibustion	Not applicable	EX-HN3, EX-HN8 (no data on side)	Not applicable	Feel the local skin warm and slightly hot without burning pain, and the local skin is slightly red without blistering	Not applicable	30–40 min	Ginger-separated moxibustion	14–70	Once or twice a day	14–70 days	None

Li and Fu [28]	Manual acupuncture	Not reported	LI20, EX-HN3, GV20, LI4, GB20, LU5, LU7 (no data on side)	LI20: 0.1–0.3 cun EX-HN3: 0.3–0.5 cun GV20, LU5: 0.5–0.8 cun LI4: 0.5–1 cun GB20: 0.8–1.2 cun LU7: 0.2–0.5 cun	Not reported	Needle manipulation every 5 min	25 min	Not reported	15	Once a day	15 days	None

Ma et al. [29]	Manual acupuncture	Not reported	ST7 (no data on side)	2.5 cun	Deqi (soreness, numbness, heaviness or distension)	Strong twirling and trembling method	No retention	3 cun filiform acupuncture needle	6	Once a week	6 weeks	6 months

Rossberg et al. [30]	Manual acupuncture	Not reported	According to the four TCM diagnosis-retention of damp in the yangming channel, damp combined with heat: (bilateral) LI4, LI11, ST40, ST44-retention of phlegm in the shaoyang channel, liver fire: (Bilateral) GB34, LR2, LI4, LR3	Facial/hand/feet: 0.5 cun arms/legs/trunk: 1.3 cun	Deqi (soreness, numbness, heaviness or distension)	Stimulated manually using reducing or reinforcing methods	25 min	0.28 mm × 25–40 mm filiform acupuncture needle	10	Not reported	4 weeks	11 months

Sun [31]	Manual acupuncture	Not reported	LI20, BL2, EX-HN5, EX-HN3, GV20, GV24, LI4, ST36 (no data on side)	Not reported	Not reported	Needle manipulation every 10 min	30 min	Not reported	Not reported	Once or twice a day (if there was no effect within 4 hours after the initial acupuncture, acupuncture was performed once more.)	Not reported	None

Zhang et al. [32]	Electroacupuncture	4	Bilateral LI4, PC6	Not reported	Not reported	Wave of condensation and rarefaction, 2/100 Hz, 8–12 mA, neutral supplementation and draining methods	30 min	Not reported	1	Once	1 day	6 hours

**Table 3:** Summary of findings.

Outcomes	Subgroup	No. of participants (RCTs)	Anticipated absolute effects (95% CI)		Relative effect (95% CI)	*I* ^2^ value (%)	Quality of evidence (grade)	Comments
			Risk with control group	Risk with treatment group				
*Acupuncture versus conventional treatment*								

TER	Total	1040 (5)	739 per 1000	865 per 1000 (776 to 961)	RR 1.17 (1.05, 1.30)	59	Moderate	Risk of bias (−1)
	Manual acupuncture	233 (3)	815 per 1000	864 per 1000 (791 to 954)	RR 1.06 (0.97, 1.17)	0	Moderate	Risk of bias (−1)
	Moxibustion	807 (2)	716 per 1000	909 per 1000 (845 to 973)	RR 1.27 (1.18, 1.36)	0	Moderate	Risk of bias (−1)

VAS	Total	253 (3)	—	MD 2.04 lower (2.1 to 1.98 lower)	—	89	Moderate	Risk of bias (−1)
	Manual acupuncture	133 (2)	—	MD 0.91 lower (1.48 to 0.34 lower)	—	65	Moderate	Risk of bias (−1)
	Moxibustion	120 (1)	—	MD 2.05 lower (2.11 to 1.99 lower)	—	NA	Moderate	Risk of bias (−1)

SNOT-20	Total (moxibustion)	120 (1)	—	MD 3.48 lower (3.58 to 3.38 lower)	—	NA	Moderate	Risk of bias (−1)

Lund-kennedy endoscopic score	Total (moxibustion)	120 (1)	—	MD 1.25 lower (1.82 to 0.68 lower)	—	NA	Moderate	Risk of bias (−1)

Lund-Mackay CT score	Total (moxibustion)	120 (1)	—	MD 1.23 lower (1.8 to 0.66 lower)	—	NA	Moderate	Risk of bias (−1)

Recurrence rate (after 6 months)	Total (manual acupuncture)	65 (1)	320 per 1000	176 per 1000 (74 to 422)	RR 0.55 (0.23, 1.32)	NA	Low	Risk of bias (−1) Imprecision (−1)

Adverse events	Total (manual acupuncture)	53 (1)	69 per 1000	41 per 1000 (4 to 432)	RR 0.60 (0.06, 6.26)	NA	Low	Risk of bias (−1) Imprecision (−1)

*Acupuncture plus conventional treatment versus conventional treatment alone*								

TER	Total	165 (2)	783 per 1000	979 per 1000 (869 to 1000)	RR 1.25 (1.11, 1.40)	0	Moderate	Risk of bias (−1)
	Manual acupuncture	100 (1)	800 per 1000	984 per 1000 (848 to 1000)	RR 1.23 (1.06, 1.41)	NA	Low	Risk of bias (−1) Imprecision (−1)
	Electroacupuncture	65 (1)	758 per 1000	970 per 1000 (788 to 1000)	RR 1.28 (1.04, 1.57)	NA	Low	Risk of bias (−1) Imprecision (−1)

VAS	Total	195 (2)	—	MD 1.07 lower (1.27 to 0.87 lower)	—	98	Moderate	Risk of bias (−1)
	Electroacupuncture	120 (1)	—	MD 0.19 higher (0.2 lower to 0.58 higher)	—	NA	Moderate	Risk of bias (−1)
	TEAS	75 (1)	—	MD 1.50 lower (1.73 to 1.27 lower)		NA	Moderate	Risk of bias (−1)

Adverse events	Total (electroacupuncture)	65 (1)	0 per 1000	0 per 1000 (0 to 0)	RR 3.09 (0.13, 73.19)	NA	Low	Risk of bias (−1) Imprecision (−1)

*Acupuncture versus sham acupuncture*								

Adverse events	Total (manual acupuncture)	42 (1)	294 per 1000	441 per 1000 (185 to 1000)	RR 1.50 (0.63, 3.53)	NA	Low	Risk of bias (−1) Imprecision (−1)

CI: confidence interval; CT: computer tomography; MD: mean difference; NA: not applicable; RCT: randomized controlled trial; RR: risk ratio; SNOT: sinonasal outcome test; TEAS: transcutaneous electrical acupoint stimulation; TER: total effective rate; and VAS: visual analogue scale.

## Data Availability

All data generated or analyzed during this study are included within the article.

## Supplementary Materials

Supplementary Materials
*Supplement 1*. Search strategies used in each database and the results. *Supplement 2*. Excluded studies after full-text review.
