# Supplementary material for: Acupuncture for the Treatment of Chronic Rhinosinusitis: A PRISMA-Compliant Systematic Review and Meta-Analysis
Source: Evid Based Complement Alternat Med. 2022 Aug 31;2022:6429836. doi: 10.1155/2022/6429836 (PMC9451955; doi:10.1155/2022/6429836)
Supplement: Supplementary Materials — Supplement 1. Search strategies used in each database and the results. Supplement 2. Excluded studies after full-text review. [file 6429836.f1.zip › 6429836.f1/Supplement 1.docx]

**Supplement 1**. Search strategies used in each database and the results

**MEDLINE via PubMed**

|  | Searches | Results |
| --- | --- | --- |
| #1 | Sinusitis[MH] OR sinusitis[TIAB] OR rhinosinusitis[TIAB] OR “rhino sinusitis”[TIAB] OR nasosinusitis[TIAB] OR “naso sinusitis”[TIAB] OR “nasal sinusitis”[TIAB] OR pansinusitis[TIAB] OR “pan sinusitis”[TIAB] OR ethmoiditis[TIAB] OR antritis[TIAB] OR sphenoiditis[TIAB] OR “sinus infection”[TIAB] OR “paranasal inflammation”[TIAB] OR “paranasal infection”[TIAB] OR (inflammation NEAR sinus)[TIAB] | 32,781 |
| #2 | Acupuncture[MH] OR "Acupuncture Therapy"[MH] OR Acupressure[MH] OR Electroacupuncture[MH] OR Auriculotherapy[MH] OR Moxibustion[MH] OR “Bee Venoms”[MH] OR Apitherapy[MH] OR Phlebotomy[MH] OR acupunct*[TIAB] OR needl*[TIAB] OR acupoint*[TIAB] OR electroacupuncture*[TIAB] OR “trigger point*”[TIAB] OR acupressure[TIAB] OR moxibustion[TIAB] OR moxa[TIAB] OR pharmacopuncture*[TIAB] OR “catgut embedding”[TIAB] OR “catgut implantation”[TIAB] OR acupotom*[TIAB] OR shiatsu[TIAB] OR shiatzu[TIAB] OR “Zhi Ya”[TIAB] OR “Chih Ya”[TIAB] OR “bee venom*”[TIAB] OR “bee sting therapy”[TIAB] OR apitoxin[TIAB] OR apitherap*[TIAB] OR phlebotom*[TIAB] OR flebotom*[TIAB] OR venesection*[TIAB] OR venipuncture*[TIAB] OR bloodletting[TIAB] OR “blood letting”[TIAB] | 188,271 |
| #3 | “randomized controlled trial”[PT] OR “controlled clinical trial”[PT] OR randomized[TIAB] OR placebo[TIAB] OR “drug therapy”[SH] OR randomly[TIAB] OR trial[TIAB] OR groups[TIAB] | 5,387,237 |
| #4 | animals[MH] NOT humans[MH] | 4,985,296 |
| #5 | #1 AND #2 AND #3 NOT #4 | **39** |

**EMBASE via Elsevier**

|  | Searches | Results |
| --- | --- | --- |
| #1 | sinusitis/exp OR sinusitis:ab,ti OR rhinosinusitis/exp OR rhinosinusitis:ab,ti OR ‘rhino sinusitis’:ab,ti OR nasosinusitis:ab,ti OR ‘naso sinusitis’:ab,ti OR ‘nasal sinusitis’:ab,ti OR  pansinusitis:ab,ti OR ‘pan sinusitis’:ab,ti OR ethmoiditis:ab,ti OR antritis:ab,ti OR sphenoiditis:ab,ti OR ‘sinus infection’:ab,ti OR ‘paranasal inflammation’:ab,ti OR ‘paranasal infection’:ab,ti OR ‘sinus inflammation’:ab,ti | 57,472 |
| #2 | acupuncture/exp OR acupressure/exp OR 'acupuncture needle'/exp OR 'acupuncture point'/exp OR electroacupuncture/exp OR ‘auricular acupuncture’/exp OR 'trigger point'/exp OR moxibustion/exp OR 'bee venom'/exp OR apitherapy/exp OR phlebotomy/exp OR bloodletting/exp OR acupunct*:ab,ti OR needl*:ab,ti OR acupoint*:ab,ti OR electroacupuncture*:ab,ti OR auriculotherapy:ab,ti OR 'trigger point*':ab,ti OR acupressure:ab,ti OR moxibustion:ab,ti OR moxa:ab,ti OR pharmacopuncture*:ab,ti OR 'catgut embedding':ab,ti OR 'catgut implantation':ab,ti OR acupotom*:ab,ti OR shiatsu:ab,ti OR shiatzu:ab,ti OR 'Zhi Ya':ab,ti OR 'Chih Ya':ab,ti OR 'bee venom*':ab,ti OR 'bee sting therapy':ab,ti OR apitoxin:ab,ti OR apitherap*:ab,ti OR phlebotom*:ab,ti OR flebotom*:ab,ti OR venesection*:ab,ti OR venipuncture*:ab,ti OR bloodletting:ab,ti OR 'blood letting':ab,ti | 277,724 |
| #3 | 'crossover procedure':de OR 'double-blind procedure':de OR 'randomized controlled trial':de OR 'single-blind procedure':de OR (random* OR factorial* OR crossover* OR cross NEXT/1 over* OR placebo* OR doubl* NEAR/1 blind* OR singl* NEAR/1 blind* OR assign* OR allocat* OR volunteer*):de,ab,ti | 2,915,171 |
| #4 | #1 AND #2 AND #3 | **55** |

**CENTRAL**

|  | Searches | Results |
| --- | --- | --- |
| #1 | MeSH descriptor: [Sinusitis] explode all trees | 1,0969 |
| #2 | (sinusitis OR rhinosinusitis OR “rhino sinusitis” OR nasosinusitis OR “naso sinusitis” OR “nasal sinusitis” OR pansinusitis OR “pan sinusitis” OR ethmoiditis OR antritis OR sphenoiditis OR “sinus infection” OR “paranasal inflammation” OR “paranasal infection” OR "sinus inflammation"):ti,ab,kw | 4,036 |
| #3 | #1 OR #2 | 4,036 |
| #4 | MeSH descriptor: [Acupuncture] explode all trees | 161 |
| #5 | MeSH descriptor: [Acupuncture Therapy] explode all trees | 5,208 |
| #6 | MeSH descriptor: [Acupressure] explode all trees | 414 |
| #7 | MeSH descriptor: [Electroacupuncture] explode all trees | 875 |
| #8 | MeSH descriptor: [Auriculotherapy] explode all trees | 249 |
| #9 | MeSH descriptor: [Moxibustion] explode all trees | 508 |
| #10 | MeSH descriptor: [Bee Venoms] explode all trees | 45 |
| #11 | MeSH descriptor: [Apitherapy] explode all trees | 29 |
| #12 | MeSH descriptor: [Phlebotomy] explode all trees | 491 |
| #13 | (acupunct* OR needl* OR acupoint* OR electroacupuncture* OR “trigger point*” OR acupressure OR moxibustion OR moxa OR pharmacopuncture* OR “catgut embedding” OR “catgut implantation” OR acupotom* OR shiatsu OR shiatzu OR “Zhi Ya” OR “Chih Ya” OR “bee venom*” OR “bee sting therapy” OR apitoxin OR apitherap* OR phlebotom* OR flebotom* OR venesection* OR venipuncture* OR bloodletting OR “blood letting”):ti,ab,kw | 38,257 |
| #14 | #4 OR #5 OR #6 OR #7 OR #8 OR #9 OR #10 OR #11 OR #12 OR #13 | 38,296 |
| #15 | (#3 AND #14) in Trials | **33** |

**AMED via EBSCO**

|  | Searches | Results |
| --- | --- | --- |
| #1 | Sinusitis[SU] OR sinusitis[TX] OR rhinosinusitis[TX] OR “rhino sinusitis”[TX] OR nasosinusitis[TX] OR “naso sinusitis”[TX] OR “nasal sinusitis”[TX] OR pansinusitis[TX] OR “pan sinusitis”[TX] OR ethmoiditis[TX] OR antritis[TX] OR sphenoiditis[TX] OR “sinus infection”[TX] OR “paranasal inflammation”[TX] OR “paranasal infection”[TX] OR “sinus inflammation”[TX] | 185 |
| #2 | Acupuncture[SU] OR "Acupuncture Therapy"[SU] OR Acupressure[SU] OR Electroacupuncture[SU] OR Auriculotherapy[SU] OR Moxibustion[SU] OR “Bee Venoms”[SU] OR Apitherapy[SU] OR Phlebotomy[SU] OR acupunct*[TX] OR needl*[TX] OR acupoint*[TX] OR electroacupuncture*[TX] OR “trigger point*”[TX] OR acupressure[TX] OR moxibustion[TX] OR moxa[TX] OR pharmacopuncture*[TX] OR “catgut embedding”[TX] OR “catgut implantation”[TX] OR acupotom*[TX] OR shiatsu[TX] OR shiatzu[TX] OR “Zhi Ya”[TX] OR “Chih Ya”[TX] OR “bee venom*”[TX] OR “bee sting therapy”[TX] OR apitoxin[TX] OR apitherap*[TX] OR phlebotom*[TX] OR flebotom*[TX] OR venesection*[TX] OR venipuncture*[TX] OR bloodletting[TX] OR “blood letting”[TX] | 14,028 |
| #3 | #1 AND #2 | **17** |

**CINAHL via EBSCO**

|  | Searches | Results |
| --- | --- | --- |
| #1 | Sinusitis[MH] OR sinusitis[TX] OR rhinosinusitis[TX] OR “rhino sinusitis”[TX] OR nasosinusitis[TX] OR “naso sinusitis”[TX] OR “nasal sinusitis”[TX] OR pansinusitis[TX] OR “pan sinusitis”[TX] OR ethmoiditis[TX] OR antritis[TX] OR sphenoiditis[TX] OR “sinus infection”[TX] OR “paranasal inflammation”[TX] OR “paranasal infection”[TX] OR “sinus inflammation”[TX] | 12,583 |
| #2 | Acupuncture[MH] OR "Acupuncture Therapy"[MH] OR Acupressure[MH] OR Electroacupuncture[MH] OR Auriculotherapy[MH] OR Moxibustion[MH] OR “Bee Venoms”[MH] OR Apitherapy[MH] OR Phlebotomy[MH] OR acupunct*[TX] OR needl*[TX] OR acupoint*[TX] OR electroacupuncture*[TX] OR “trigger point*”[TX] OR acupressure[TX] OR moxibustion[TX] OR moxa[TX] OR pharmacopuncture*[TX] OR “catgut embedding”[TX] OR “catgut implantation”[TX] OR acupotom*[TX] OR shiatsu[TX] OR shiatzu[TX] OR “Zhi Ya”[TX] OR “Chih Ya”[TX] OR “bee venom*”[TX] OR “bee sting therapy”[TX] OR apitoxin[TX] OR apitherap*[TX] OR phlebotom*[TX] OR flebotom*[TX] OR venesection*[TX] OR venipuncture*[TX] OR bloodletting[TX] OR “blood letting”[TX] | 144,751 |
| #3 | #1 AND #2 | **1,236** |

**OASIS**

|  | Searches | Results |
| --- | --- | --- |
| #1 | (부비동염\|비부비동염\|부비강염\|축농증\|코곁굴염) (침\|혈위\|경혈\|지압\|뜸\|봉독\|사혈) | **0** |

**KMbase**

|  | Searches | Results |
| --- | --- | --- |
| #1 | (((([ALL=부비동염] OR [ALL=비부비동염]) OR [ALL=부비강염]) OR [ALL=축농증]) OR [ALL=코곁굴염]) | 530 |
| #2 | (((((([ALL=침] OR [ALL=혈위]) OR [ALL=경혈]) OR [ALL=지압]) OR [ALL=뜸]) OR [ALL=봉독]) OR [ALL=사혈]) | 14,549 |
| #3 | #1 AND #2 | **55** |

**KISS**

|  | Searches | Results |
| --- | --- | --- |
| #1 | 초록=(부비동염\|비부비동염\|부비강염\|축농증\|코곁굴염) AND 초록=(침\|혈위\|경혈\|지압\|뜸\|봉독\|사혈) | **34** |

**ScienceON**

|  | Searches | Results |
| --- | --- | --- |
| #1 | (부비동염\|비부비동염\|부비강염\|축농증\|코곁굴염) (침\|혈위\|경혈\|지압\|뜸\|봉독\|사혈) | **9** |

**RISS**

|  | Searches | Results |
| --- | --- | --- |
| #1 | (부비동염\|비부비동염\|부비강염\|축농증\|코곁굴염) (침\|혈위\|경혈\|지압\|뜸\|봉독\|사혈) | **8** |

**CNKI**

|  | Searches | Results |
| --- | --- | --- |
| #1 | (SU='鼻窦炎'+'窦炎'+'鼻渊'+‘脑漏'+‘鼻道炎'+'鼻痈'+'蓄脓症'+'蓄脓'+'副鼻腔炎') AND (SU='针'+'刺法'+'灸'+'埋线'+'蜂毒'+'蜂疗'+'药物穿刺'+'穴位'+'腧穴'+'指压'+'放血'+'刺络') AND (SU='随机'+'对照'+'随意'+'试验'+'安慰') | **22** |

**Wanfang data**

|  | Searches | Results |
| --- | --- | --- |
| #1 | (主题:鼻窦炎 OR 主题:窦炎 OR 主题:鼻渊 OR 主题:脑漏 OR 主题:鼻道炎 OR 主题:鼻痈 OR 主题:蓄脓症 OR 主题:蓄脓 OR 主题:副鼻腔炎) AND (主题:针 OR 主题:刺法 OR 主题:灸 OR 主题:埋线 OR 主题:蜂毒 OR 主题:蜂疗 OR 主题:药物穿刺 OR 主题:穴位 OR 主题:腧穴 OR 主题:指压 OR 主题:放血 OR 主题:刺络) AND (主题:随机 OR 主题:对照 OR 主题:随意 OR 主题:试验 OR 主题:安慰) | **919** |

**VIP.**

|  | Searches | Results |
| --- | --- | --- |
| #1 | (M=(鼻窦炎 OR 窦炎 OR 鼻渊 OR 脑漏 OR 鼻道炎 OR 鼻痈 OR 蓄脓症 OR 蓄脓 OR 副鼻腔炎) AND M=(针 OR 刺法 OR 灸 OR 埋线 OR 蜂毒 OR 蜂疗 OR 药物穿刺 OR 穴位 OR 腧穴 OR 指压 OR 放血 OR 刺络) AND M=(随机 OR 对照 OR 随意 OR 试验 OR 安慰)) | **3** |

**CiNii**

|  | Searches | Results |
| --- | --- | --- |
| #1 | (副鼻腔炎 OR 蓄膿症 OR じょうみゃくどうえん OR せいふくびくうえん OR 上顎洞炎 OR じょうがくどうえん OR 前頭洞炎 OR ぜんとうどうえん OR 静脈洞炎) AND (針 OR 鍼 OR 灸 OR 蜂毒 OR 蜂療法 OR 蜂針療法 OR 穴位注射 OR 穴 OR 壺 OR 圧痛点 OR 指圧 OR 瀉血 OR 刺絡) AND (ランダム化比較試験 OR 対照臨床試験 OR ランダム OR 無作為 OR 対照 OR 試験 OR 偽薬) | **3** |
