# Supplementary material for: Acupuncture for the Treatment of Chronic Rhinosinusitis: A PRISMA-Compliant Systematic Review and Meta-Analysis
Source: Evid Based Complement Alternat Med. 2022 Aug 31;2022:6429836. doi: 10.1155/2022/6429836 (PMC9451955; doi:10.1155/2022/6429836)
Supplement: Supplementary Materials — Supplement 1. Search strategies used in each database and the results. Supplement 2. Excluded studies after full-text review. [file 6429836.f1.zip › 6429836.f1/Supplement 2.docx]

**Supplement 2. Excluded studies after full-text review**

**1) not RCT: 10**

1. 刘广霞, 邢春光. 针灸治疗鼻渊临床研究. 针刺研究. 2000;25(2):141-3.

2. 王庆先, 罗勋旺, 龙天贵. 穴位割治治疗鼻渊125例临床观察. 第三届兰茂论坛暨2016年云南省中医药界学术年会论文集; 昆明2016. p. 413-4.

3. 刘利敏. 穴位透刺联合鼻窦灌注液治疗慢性鼻窦炎效果观察. 中国疗养医学. 2020;29(05):526-8.

4. 李惠君, 吕楠, 王晓梅. 不同中医护理干预方式对鼻窦炎术后患者疼痛的影响评价. 母婴世界. 2017(20):134.

5. 辛晓艳, 程梅, 肖秀英, 邓少颜. 不同中医护理干预方式对鼻窦炎术后患者疼痛的影响. 护理与康复. 2015;14(8):756-8.

6. 刘彩梅. 针刺蝶腭神经节治疗鼻渊76例. 现代中医药. 2009;29(6):51-.

7. 卢嫏环, 谢强. 谢氏围手术期中医平衡康复疗法对慢性鼻-鼻窦炎围手术期干预的临床观察. 时珍国医国药. 2014;25(12):2947-8.

8. Kim JI, Choi JY, Lee MS, Kim TH, Kim AR, Jung SY, et al. Acupuncture for improving chronic rhinosinusitis complicated with persistent allergic rhinitis. Forschende Komplementarmedizin. 2010;17(6):333-5.

9. 汪玉娇. 《鼻炎穴位按摩保健操》改善鼻炎患者通气状况及预防慢性鼻—鼻窦炎急性发作的“治未病”相关研究 [硕士]: 北京中医药大学; 2013.

10. 郑开运. 蜂针疗法治疗鼻炎鼻窦炎的临床应用. 中国养蜂. 2002(03):17.

**2) not about chronic rhinosinusitis: 6**

1. 程梅, 肖秀英, 刘谦虚, 邓少颜, 罗瑞玲, 辛晓艳, et al. 穴位按摩加耳穴贴压缓解鼻窦炎患者术后疼痛的研究. 护理学报. 2014(9):65-7.

2. 晏英. 胆经穴位贴片结合西药治疗急性上颌窦炎60例临床研究 [硕士]: 贵阳中医学院; 2009.

3. 魏宏珊, 生兆梅, 刘艳, 赵慧萍, 郝维强, 党金军. 基于中医护理干预探讨穴位按摩联合耳穴贴压对鼻窦炎患者疗效及安全性的临床研究. 系统医学. 2020;5(18):141-3,71.

4. 王茂珩. 改良隔姜灸联合罗红霉素治疗鼻渊临床体会. 亚太传统医药. 2016;12(8):89-90.

5. 朱喜艳, 张伟. 穴位按摩与耳穴贴压对鼻窦炎患者临床护理效果及安全性分析. 新疆中医药. 2021;39(06):46-47.

6. 李静林, 蒋洁丽, 朱丽安, 谢慧娴. 腕踝针治疗对缓解患者鼻部术后伤口疼痛的临床效果. 当代护士（上旬刊）. 2021;28(2):113-114.

**3) not about only acupuncture: 8**

1. Chen G. Application effects of acupuncture anesthesia and local anesthesia to radiofrequency ablation surgery for chronic nasosinusitis. Western Journal of Traditional Chinese Medicine. 2019;32(3):112-4.

2. Cui N, Lei Q. Analgesic effect of finger pressing and massaging for children after endoscopic nasal sinus operation. Journal of Nursing Science. 2012;27(16):45-6.

3. 李雪梅. 中医护理干预对慢性鼻窦炎围手术期的效果分析. 养生保健指南. 2018(8):203.

4. 祁顺来, 朵德龙. 中医辨证施治联合鼻喷糖皮质激素治疗慢性鼻—鼻窦炎伴鼻息肉的临床疗效. 中国保健营养. 2020;30(25):57.

5. 耿银娣. 中西医结合治疗过敏性鼻窦炎的效果观察. 医学信息. 2013(25):501-2.

6. 毛承深, 胡娟. 中西医结合治疗慢性鼻窦炎178例疗效观察. 中国中西医结合杂志. 2001;21(9):712-3.

7. 张健, 茅金金. 鼻窦炎围手术期中西医康复方案应用对预后影响分析. 医学信息. 2014(16):67.

8. 周利君, 韦燕萍, 陈贻芳, 谭伊婷. 鼻窦炎鼻内镜术后患者睡眠障碍原因分析及护理对策综合分析. 东方药膳. 2021;(9):184.

**4) using other CIM intervention in control group: 3**

1. 丁会军. 针药并施治疗副鼻窦炎疗效观察. 北京针灸骨伤学院学报. 2000;0(1).

2. 尚国超, 马小闵. 鼻渊通窍颗粒联合针灸对鼻窦炎患者的临床疗效. 中成药. 2021;43(4):1114-1116.

3. 丁芬, 王成云. 温针灸结合中药治疗慢性鼻窦炎临床分析. 实用中医药杂志. 2021;37(5):750-751.

**5) duplicate data: 3**

1. Guo R. Traditional Chinese acupuncture for chronic sinusitis: is it as good as conventional treatment or no better than sham acupuncture? Focus on Alternative & Complementary Therapies. 2005;10(4):303-4.

2. Stavem K, Rossberg E, Larsson PG. Health-related quality of life outcomes in a trial of acupuncture, sham acupuncture and conventional treatment for chronic sinusitis. BMC research notes. 2008;1(1):37.

3. Liu LZ, Guo DW, Liang ML, He ZQ. Clinical observation on treatment of 60 cases of chronic rhinosinusitis with ginger-separated moxibustion. Hunan Journal of Traditional Chinese Medicine. 2020;36(8):80-2.
